# Transcriptomic Profiling of *Gossypium barbadense* Under *Fusarium oxysporum* f. sp. *vasinfectum* Race 7 Infection via Bulk RNA-Seq and snRNA-Seq

**DOI:** 10.3390/cells15211935

**Published:** 2026-10-24

**Authors:** Baojun Liu, Gang Liu, Jianyu Bai, Yujia Zhang, Shu Wang, Aixing Gu, W. G. Dilantha Fernando

**Affiliations:** 1 College of Agriculture, Xinjiang Agricultural University, Urumqi 830052, Chinaliu09997776@163.com (G.L.);; 2 Institute of Forest Ecology and Landscaping, Xinjiang Academy of Forestry Sciences, Urumqi 830063, China; 3 Engineering Research Centre of Cotton, Ministry of Education, Urumqi 830052, China; 4 Department of Plant Science, University of Manitoba, Winnipeg, MB R3T 2N2, Canada

**Keywords:** Cotton, *Gossypium barbadense*, *Fusarium oxysporum*, RNA-seq, snRNA-seq

## Abstract

**Highlights:**

**What are the main findings?**

- Time-course bulk RNA-seq showed that the strongest transcriptional response occurred at 2 hpi (9230 DEGs), and integration of K-means cluster C4 with the WGCNA turquoise module identified 1503 core early-defense genes enriched in JA/ABA signaling, wounding response, and defense regulation.
- snRNA-seq resolves 32,773 root nuclei and identifies root cap and epidermis cells as the primary early immune responders, with pseudotime trajectories revealing ordered defense activation.

**What are the implications of the main findings?**

- This study provides a citable cellular-resolution transcriptomic resource for cotton–Fov7 interaction and root immunity research.
- The identified cell types and candidate genes provide targets for functional validation and molecular breeding of Fusarium wilt-resistant cotton.

**Abstract:**

Cotton, as an economically important crop, suffers significant yield losses from infection by *Fusarium oxysporum* f. sp. *vasinfectum* race 7 (Fov7) infection. To elucidate the molecular mechanisms underlying host responses to Fov7 infection, we conducted a comprehensive transcriptional analysis of *Gossypium barbadense* using integrated bulk RNA sequencing (RNA-seq) and single-nucleus RNA sequencing (snRNA-seq) approaches. Our time-course study encompassed four critical infection stages (0, 2, 12, and 48 h post-inoculation) to characterize spatiotemporal transcriptomic dynamics. Bulk RNA-seq analysis revealed that the number of differentially expressed gene (DEG) numbers peaked at 2 h post-inoculation (hpi), identifying 9230 DEGs and demonstrating substantial transcriptional activation during early pathogen challenge. K-means clustering classified DEGs into six temporal expression patterns, with cluster C4 showing significant enrichment of defense-related pathways. Weighted gene co-expression network analysis (WGCNA) identified ten distinct co-expression modules, among which the turquoise module exhibited strong correlation with early infection responses. Integration of K-means clustering and WGCNA results pinpointed 1503 key defense-related genes. A single-nucleus atlas of cotton roots during Fov7 infection was constructed, comprising 32,773 nuclei, which revealed the cellular heterogeneity of cotton root tips. Functional enrichment analysis using these key genes emphasized the crucial roles of root cap and epidermal cells in pathogen perception and defense activation. Pseudotime trajectory mapping further delineated transcriptional dynamics in these cell populations during infection progression, demonstrating pathway activation in the root cap and epidermis at later infection stages. This multi-omics study establishes a valuable transcriptional resource for dissecting cotton’s resistance mechanisms against Fov7 and provides theoretical insights for developing disease-resistant cultivars through molecular breeding strategies.

## 1. Introduction

Cotton (Gossypium species) represents one of the most significant economic crops globally. It is cultivated in approximately 80 countries and accounts for about 2.5% of the world’s arable land, generating an estimated annual revenue of around 500 billion dollars [1,2,3]. Among cotton species, Gossypium barbadense is highly valued in the textile industry due to its superior fiber quality and characteristics, thereby maintaining a dominant position in the high-end textile raw material market [2,3,4,5]. However, the productivity and quality of *G. barbadense* face severe threats from *Fusarium oxysporum* f. sp. *vasinfectum* Race (Fov), a widespread soil-borne pathogen capable of infecting diverse host plants, including cotton, watermelon and banana [6,7]. This pathogen invades cotton seedlings through root tips, causing characteristic symptoms such as leaf wilting and necrosis [8,9]. Eight races of Fov have been identified worldwide [3,10]. In China, three races (3, 7, and 8) of Fov have been reported, with race 7 being the most widely distributed and having the highest virulence in China [8,11]. Despite the development of several resistant cotton cultivars, current understanding remains limited regarding the molecular mechanisms underlying Fov resistance and its genetic basis.

In recent years, breakthroughs in single-cell RNA sequencing technology have provided a revolutionary tool for deciphering cellular heterogeneity in plants [12], and it has been extensively applied in many studies of pathogen infections across a variety of plant species. For instance, single-cell transcriptomics has uncovered cell-type-specific responses to fungal infection in Arabidopsis thaliana leaves infected with Colletotrichum higginsianum [13]. In garlic, a single-cell transcriptomic atlas enabled the first characterization of cell differentiation during bulb enlargement and uncovered cell-type-specific responses in virus–host interactions [14]. In maize, Single-cell transcriptomic profiling of leaves revealed leaf cell heterogeneity and systemic immune responses against Puccinia polysora Underw [2]. In tobacco, single-nucleus transcriptome analysis revealed the cellular and molecular responses of roots to Ralstonia solanacearum invasion [3]. Multiple single-cell studies have been conducted in cotton [15], focusing on diverse biological processes, including fiber development [16,17,18], anther development [19], leaf terpenoid biosynthesis [20], embryogenesis and plant regeneration [21,22]. Recently, single-cell sequencing has also been applied to investigate salt stress responses in diploid cotton roots [23]. However, no studies have yet reported single-cell transcriptomic analyses of tetraploid cotton roots during pathogen infection.

This study systematically characterizes the transcriptional dynamics of Gossypium barbadense in response to Fov7 infection through integrated analysis of bulk RNA sequencing (RNA-seq) and single-nucleus RNA sequencing (snRNA-seq) technologies. The study covers four key time points (0, 2, 12, and 48 h post-inoculation) to elucidate the spatiotemporal transcriptional dynamics in cotton during pathogen invasion, thereby establishing a multi-scale transcriptional atlas that enhances understanding of molecular resistance mechanisms against Fov7 and provides a theoretical framework for disease-resistant crop breeding.

## 2. Materials and Methods

### 2.1. Plant Materials and Cultivation System

The *Fusarium oxysporum* f. sp. *vasinfectum* race 7 (Fov7) resistant Sea Island cotton (Gossypium barbadense L.), variety “06-146(RC)”, was used in this study, provided by the Crop Genetics and Breeding Laboratory of Xinjiang Agricultural University. Seeds were surface-sterilized with 0.1% HgCl_2_ and germinated in a constant temperature incubator (Ningbo Jiangnan Instrument Factory, Ningbo, China; 28 ± 0.5 °C). Once primary roots reached 3 cm in length, seedlings were transferred to a hydroponic system containing Hoagland’s nutrient solution (pH 6.0 ± 0.2). Plants were grown under controlled conditions with a 16 h/8 h (day/night) photoperiod, light intensity of 100 μmol·m^−2^·s^−1^ (LED full-spectrum light source), relative humidity of 65 ± 5%, and constant temperature of 28 ± 1 °C.

### 2.2. Pathogen Preparation and Inoculation

The highly virulent *Fusarium oxysporum* f. sp. *vasinfectum* race 7 isolate st89 was obtained from the Plant Pathology Laboratory, Xinjiang Agricultural University, China. The isolate was activated as follows: (1) frozen fungal isolates were cultured on PDA solid medium (containing 50 μg/mL chloramphenicol) at 25 °C in darkness for 5 days; (2) mycelial plugs from the colony edge were transferred to PDB liquid medium (without agar) and incubated in a shaking incubator (Shanghai Tiancheng Experimental Instrument Manufacturing Co., Ltd., Shanghai, China; 25 ± 0.5 °C, 180 rpm) for 7–10 days to obtain high-purity conidial suspensions. The spore concentration was adjusted to 1 × 10^8^ spores/mL using a hemocytometer (Changsha Xiangbo Scientific Instrument Co., Ltd., Changsha, China), and 0.01% Tween-20 was added as a surfactant.

### 2.3. Experimental Design and Sample Collection

When cotton seedlings developed two fully expanded true leaves (14 ± 1 days after sowing), pathogen inoculation was performed using the root irrigation method (5 mL inoculum per plant). Root tissue samples were collected at 0, 2, 12, and 48 h post-inoculation (hpi), immediately flash-frozen in liquid nitrogen, and stored at −80 °C until further analysis. Each biological replicate consisted of pooled root tissue from five independent seedlings, and three biological replicates were collected at each time point. The pooled root tissues were aliquoted for bulk RNA sequencing and single-nucleus RNA sequencing (snRNA-seq), ensuring that both datasets were derived from the same batch of inoculated seedlings and the same inoculation time course. For snRNA-seq, two of the three biological replicates were used at each time point, resulting in a total of 8 snRNA-seq samples. For bulk RNA sequencing, all three biological replicates were used at each time point, producing a total of 12 samples.

### 2.4. RNA-Seq Library Preparation and Analysis

Following RNA-seq library construction protocols [24], libraries were prepared from samples collected at four key time points (0 hpi, 2 hpi, 12 hpi, and 48 hpi), with three biological replicates per time point, and then sent to Genepioneer Biotechnologies (Nanjing, China) for RNA sequencing. Paired-end sequencing (150 bp) was carried using an Illumina NovaSeq 6000 system (Illumina Inc., San Diego, CA, USA). A total of 278,954,981 high-quality reads were obtained across all samples, with the average Q20 and Q30 values being 98.88% and 96.83%, respectively, and the Q30 of all samples being 95%, demonstrating successful RNA-seq library preparation and sequencing (Table 1). After sequencing, low-quality sequences and adapter sequences were removed, and clean reads were aligned to the Gossypium barbadense H7124_ZJU genome [25] using HISAT2 2.2.1 [26] with default parameters. StringTie v2.2.0 [27] was used to derive per-gene read counts, and gene expression levels were quantified as FPKM (Fragments Per Kilobase of transcript per Million mapped reads). Differentially expressed genes (DEGs) were identified with the R package DESeq2 (v1.26.0) [28] using the raw integer read counts obtained from StringTie (rather than FPKM values), with a design formula of ~ time_point (0, 2, 12, and 48 hpi). Pairwise comparisons were performed for each infected time point against the 0 hpi control (i.e., 2 vs. 0, 12 vs. 0, and 48 vs. 0 hpi) using Wald tests, and genes were considered DEGs when |log_2_ fold change| > 1 and the Benjamini–Hochberg-adjusted false discovery rate (FDR) was <0.05. Because all 12 libraries were prepared and sequenced in a single experimental batch, no batch term was included in the design; the tight clustering of biological replicates and clear separation of samples by time point on principal component analysis (PCA) confirmed the absence of batch effects (Supplementary Figure S1). FPKM values were used solely for expression visualization (heatmaps and line plots) and as the input for K-means clustering. To further resolve the temporal dynamics of gene expression and identify functionally coherent gene groups, we performed time-series K-means clustering using the R package ClusterGVis [29]. Furthermore, we used the R package clusterProfiler v4.14.4 to perform GO enrichment analysis [30]. The gene co-expression network was constructed by the R package WGCNA (v1.74) [31], which was used to identify modules with highly similar expression patterns. The following fitting parameters were employed: soft threshold = 10, minimum module size = 30, merging cut height = 0.20.

### 2.5. Single-Nucleus RNA Library Preparation and Quality Control

Two biological replicates were collected at each time point, resulting in a total of eight snRNA-seq libraries. All procedures for preparing high-quality single-nucleus suspensions were performed using a clean bench. Fresh root tip tissue (approximately 1 cm in length) was placed in a culture dish, covered with 1–2 mL pre-cooled nuclei isolation buffer A (0.1 mM DTT, 0.1 mM PMSF, and 0.4 U/μL RNase inhibitor), and chopped slowly with a sharp blade to approximately 1 mm^3^ on ice, followed by incubation for 5 min. The tissue suspension was filtered through a 30 μm mesh, and the filtrate was collected. Buffer A was added to the filtrate to a final volume of 5 mL, followed by incubation on ice for 5 min. The sample was centrifuged at 50× *g* for 5 min at 4 °C, and the supernatant was collected. The supernatant was then centrifuged at 2000× *g* for 10 min at 4 °C, and the pellet was retained. The pellet was gently resuspended in 1–2 mL washing buffer A by pipetting up and down 15 times. In a 15 mL centrifuge tube, 25% isolation buffer B (nuclei isolation buffer B:PBS = 25:75) and 75% isolation buffer B (nuclei isolation buffer B:PBS = 75:25) were sequentially added, followed by slow addition of the resuspended nuclei (volume ratio 1:1:1). The tube was centrifuged at 3000× *g* for 15 min at 4 °C with the acceleration/deceleration set to level 4, and the middle layer (approximately 1 mL) was collected. Three volumes of washing buffer B were added and mixed, followed by centrifugation at 1800× *g* for 10 min at 4 °C. The pellet was resuspended in PBS containing 1% BSA and gently mixed. For quality assessment, 10 μL of the nuclei suspension was mixed with 10 μL of 100× DAPI and incubated for 5 min in the dark. A 7–10 μL aliquot was loaded onto a hemocytometer, and blue fluorescent nuclei in four large squares were counted under a fluorescence microscope (Nikon Corporation, Tokyo, Japan;10×). Nuclear integrity was assessed by trypan blue staining; the proportion of intact nuclei was required to be ≥90%, and the nuclei concentration was adjusted to 1 × 10^6^–5 × 10^6^ nuclei/mL using a hemocytometer before downstream use. Qualified single-nucleus suspensions were loaded onto a MobiNova-100 high-throughput single-cell controller (MobiDrop, Zhejiang, China), and single-nucleus RNA-seq libraries were constructed using the MobiCube RNA-seq Single Cell Kit (MobiDrop). The workflow included nucleus lysis and RNA release, oligo-dT-primed first-strand cDNA synthesis, cDNA amplification (15 cycles), and library fragmentation and adapter ligation. Library quality was assessed with an Agilent 2100 Bioanalyzer (Agilent Technologies Inc., Santa Clara, CA, USA), showing a main peak at 300–500 bp, and concentrations quantified by Qubit 4.0 (Thermo Fisher Scientific, Waltham, MA, USA) were all ≥2 nM. Sequencing was performed on the MobiNova-100 platform using the Illumina NovaSeq 6000 paired-end strategy; Read1 (28 bp) captured barcodes and UMIs, and Read2 (150 bp) captured RNA sequences (Table 2). Low-quality reads (reads with >50% of bases having a Phred score < 20), poly-A-containing redundant sequences, and adapter-contaminated sequences were removed to obtain clean reads. Clean data were processed using the MobiVision platform (https://www.mobidrop.com/bioinformatics/mobivision2; accessed on 20 March 2026) for barcode demultiplexing, UMI count correction and construction of gene expression matrices were based on the Gossypium barbadense H7124_ZJU reference genome annotation. Downstream quality control and normalization were performed using Seurat v4.4.0 [32]. Nuclei with UMI counts <500 (likely ruptured nuclei or empty droplets) or >6000 (suspected doublets or aggregated nuclei) were discarded; these thresholds were set based on the UMI distribution of normal root-tip nuclei in preliminary experiments (median ± 2 standard deviations). Genes detected in fewer than 3 nuclei were excluded. Only nuclei with a mitochondrial gene transcript proportion <10% were retained. In addition, DoubletFinder (v2.0.2) was used to identify doublets by comparing the simulated expected doublet rate (set at 5%) with the observed data; suspected doublets were flagged and removed, and only nuclei labeled as “single cells” were retained [33]. A total of 32,773 nuclei were obtained (Table 3), with mean unique reads per nucleus ranging from 1460 to 2296.

### 2.6. Nucleus Clustering, Annotation and Pseudotime Trajectory Analysis

We performed an in-depth analysis of cotton root tip single-nucleus RNA sequencing data, focusing on systematic nucleus clustering and cell type annotation. To integrate single-nucleus datasets from various time points, we employed Canonical Correlation Analysis (CCA). By leveraging the Seurat v4.4.0 package [34], we effectively mitigated batch effects while preserving genuine biological variations. Data normalization was conducted using the LogNormalize method (scale factor = 10,000), followed by log1p transformation. We identified 2000 highly variable genes using the “FindVariableGenes” function (vst method) and scaled the data using the “ScaleData” function. Principal component analysis (PCA) was performed using the “RunPCA” function in Seurat, selecting 40 principal components with a random seed set to 42 for dimensionality reduction. Graph-based cell clustering was then conducted using the Leiden algorithm (resolution = 0.5). Nonlinear dimensionality reduction visualization was achieved through the RunUMAP function (dims = 1:40). For cell type annotation, we integrated known marker gene information from multiple plant single-cell databases, including PlantscRNAdb and scPlantDB [35,36]. We performed trajectory analyses using Monocle2 to track their progression through the infection process [37].

### 2.7. Quantitative Real-Time PCR Validation

Total RNA was isolated from experimental samples, with integrity verified via agarose gel electrophoresis and concentration/purity quantified on an ND5000 NanoDrop UV–Vis spectrophotometer (Thermo Fisher Scientific Inc., USA). First-strand cDNA was synthesized from 200 ng of total RNA using the Hifair™ III 1st Strand cDNA Synthesis SuperMix for qPCR (YEASEN). Gene-specific primers were designed with Primer Premier 5.0 software, and their full sequences are provided in Supplementary Table S1. qRT-PCR was performed on an ABI 7500 instrument (Applied Biosystems, San Francisco, CA, USA) in a 20 μL reaction system containing 10.0 μL of SYBR Premix Ex Taq (2×), 1.0 μL of forward primer (10 μM), 1.0 μL of reverse primer (10 μM), and 1.0 μL of template cDNA, with Nuclease-free Water added to 20 μL. The two-step amplification conditions were as follows: 95 °C for 5 min for initial denaturation, followed by 40 cycles of 95 °C for 10 s and 60 °C for 30 s. Melting curve analysis was performed at 95 °C for 15 s, 60 °C for 60 s, and 95 °C for 15 s, with continuous fluorescence signal acquisition. Relative transcript expression levels were calculated using the 2^−ΔΔCt^ method, with *GAPDH* as the endogenous reference gene [38]. Data visualization was conducted with GraphPad Prism version 10.1.2. One-way ANOVA followed by Dunnett’s multiple comparison test was used. All experiments comprised three independent biological replicates, each with three technical replicates.

## 3. Results

### 3.1. K-Means Clustering and WGCNA Analysis DEGs Using Bulk RNA-Seq

**Figure 1:**
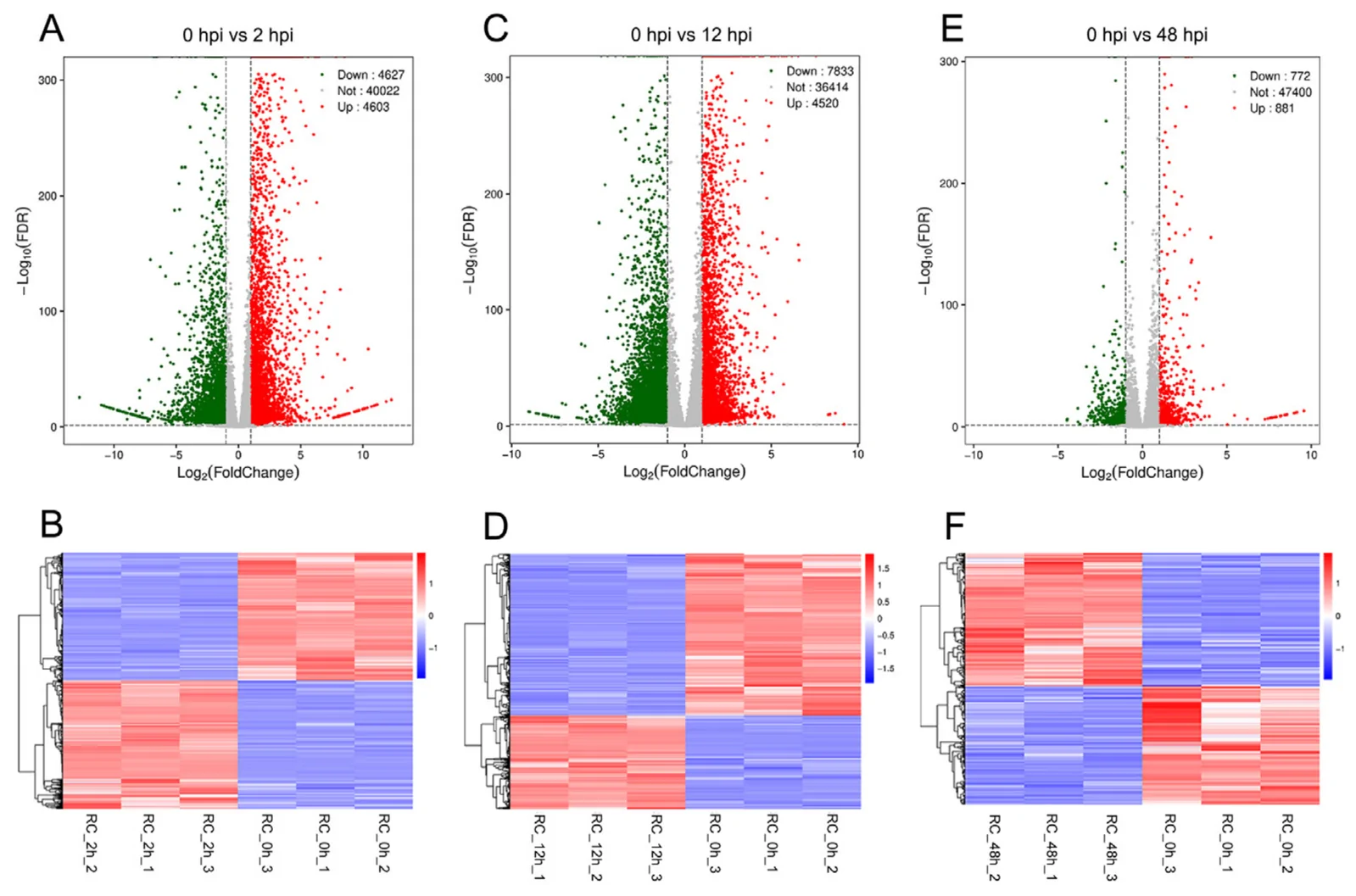
Transcriptional responses of Gossypium barbadense roots to Fov7 infection. (**A**,**B**) Volcano plot and heatmap showing differential gene expression analysis between early infection stage (2 hpi) and uninfected control. (**C**,**D**) Volcano plot and heatmap showing differential gene expression analysis between intermediate infection stage (12 hpi) and uninfected control. (**E**,**F**) Volcano plot and heatmap showing differential gene expression analysis between late infection stage (48 hpi) and uninfected control. In volcano plots, upregulated genes are shown in red, downregulated genes in blue, and non-significant genes in gray. Heatmaps display the expression patterns of DEGs with hierarchical clustering of genes.

**Figure 2:**
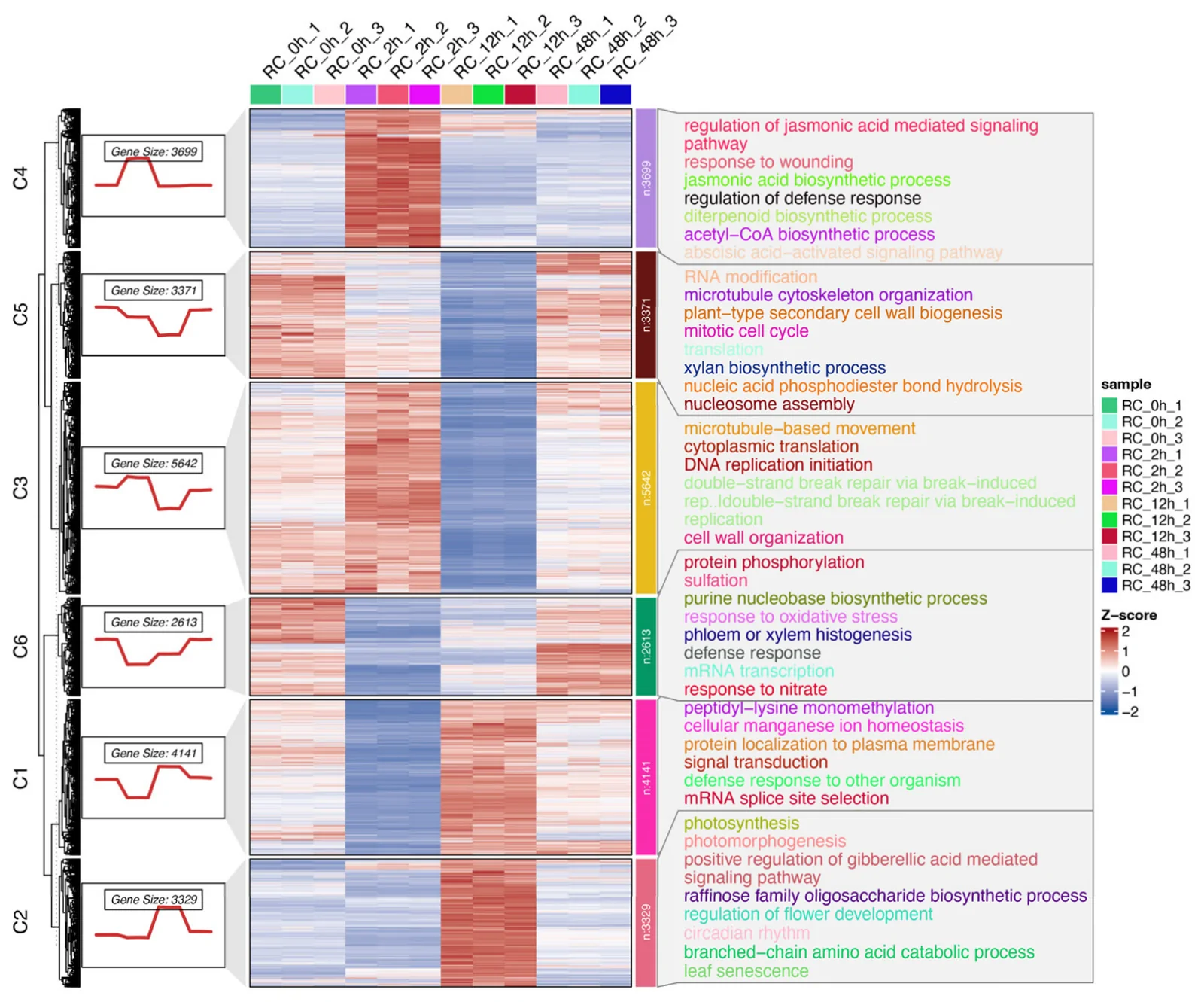
K-means clustering analysis of temporal gene expression patterns during Fov7 infection. K-means clustering partitioned all differentially expressed genes into six distinct clusters (C1–C6) based on their temporal expression profiles across four time points (0, 2, 12, and 48 hpi). Each cluster exhibits unique temporal dynamics, with line plots showing mean expression levels (FPKM). The significant results of the GO Biological Process enrichment are listed on the right side.

The high quality of the bulk RNA-seq data is underscored by the Q30 base percentage, which exceeded 96% across all 12 samples (Table 1). At the early infection stage (2 hpi), the most pronounced transcriptional changes were observed, with 9230 DEGs identified, comprising 4603 upregulated and 4627 downregulated genes (Figure 1A,B). This substantial transcriptional reprogramming indicates an immediate and robust cellular response to fungal invasion. During the intermediate stage (12 hpi), while the magnitude of gene expression changes decreased compared to 2 hpi, significant differential expression was still apparent (Figure 1C,D). Notably, the number of downregulated genes increased to 7833, potentially reflecting metabolic reprogramming and resource reallocation during sustained defense responses. At the late stage (48 hpi), gene expression patterns stabilized, with a marked reduction in DEGs to 1653 (Figure 1E,F), suggesting that the plant may have gradually adapted to pathogen infection or established a new transcriptional equilibrium. All DEGs were successfully partitioned into six distinct clusters, each exhibiting unique temporal expression patterns (Figure 2). GO enrichment for genes in each cluster and identified gene modules in cluster C4 were significantly upregulated at 2 hpi and significantly enriched for regulation of the jasmonic acid-mediated signaling pathway, response to wounding, regulation of defense response, and the abscisic acid-activated signaling pathway, further validating the functional coherence of this gene cluster and its importance in early defense responses.

**Figure 3:**
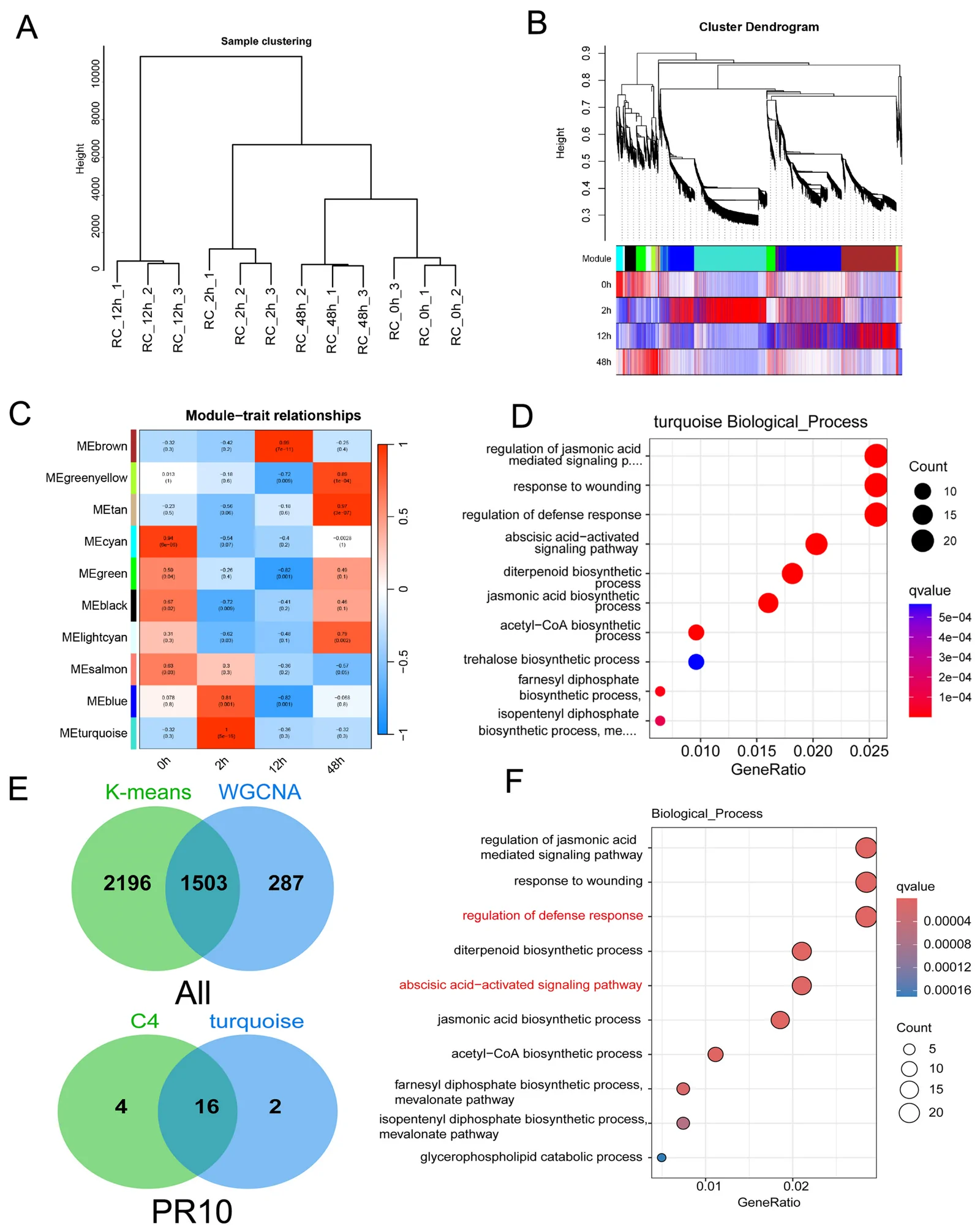
Weighted Gene Co-expression Network Analysis (WGCNA) of Gossypium barbadense response to Fov7 infection. (**A**) Hierarchical clustering dendrogram of samples. (**B**) Gene dendrogram and module assignment using dynamic tree cutting, identifying ten distinct co-expression modules represented by different colors. (**C**) Module-trait correlation heatmap showing the relationship between gene modules and infection time points. Correlation values and *p*-values are indicated. (**D**) Gene Ontology enrichment analysis of the turquoise module, displaying significantly enriched biological processes related to stress responses and defense mechanisms. (**E**) Venn diagram showing the intersection between K-means cluster C4 and WGCNA turquoise module. PR10 (Pathogenesis-Related protein 10) gene family members for two-gene-set interaction is also listed below. (**F**) Gene Ontology enrichment analysis of the 1503 intersected genes.

Sample clustering analysis demonstrated excellent reproducibility, with biological replicates clustering tightly together and samples grouping according to their respective time points (Figure 3A). Ten distinct gene modules were identified by WGCNA analysis, each represented by a unique color (Figure 3B). Module-trait correlation analysis revealed that the turquoise module showed the highest correlation with the 2 hpi treatment (Figure 3C), validating its biological relevance to early infection responses. GO enrichment analysis of the turquoise module confirmed its involvement in stress-related pathways (Figure 3D), providing biological validation for the network construction approach. To verify that the identified co-expression modules were robust rather than sample-specific, we performed two independent stability analyses. First, a bootstrap resampling procedure (30 iterations, resampling samples with replacement) was used to assess the reproducibility of module membership; for the turquoise module—the largest and most functionally relevant module—the mean Jaccard similarity of gene membership across bootstrap replicates was 0.648 (SD = 0.159), indicating largely stable membership. Second, a split-half module-preservation analysis (modulePreservation) was applied, in which the module structure in one half of the samples was tested against permuted null networks in the other half; the turquoise module was strongly preserved (Zsummary = 54.8; density Z = 62.3; connectivity Z = 47.3), far exceeding the conventional Zsummary > 10 threshold for strong preservation. Together, these results demonstrate that the identified modules, and the turquoise module in particular, are reproducible and do not represent artifacts of the particular sample set.

The intersection analysis between K-means cluster C4 and the WGCNA turquoise module identified 1503 key genes, and they specifically upregulated at the 2 hpi stage (Figure 3E). GO enrichment analysis of these intersected genes confirmed their involvement in regulation of defense response and the abscisic acid-activated signaling pathway (Figure 3F). We focused on the PR10 (Pathogenesis-Related protein 10) gene family, which is well-established for its role in plant defense responses [39], and identified 16 PR10 gene members within the early response gene set (Figure 3E).

### 3.2. Construction of the Cotton Root Single-Nucleus Transcriptome Atlas During Fov7 Infection

**Figure 4:**
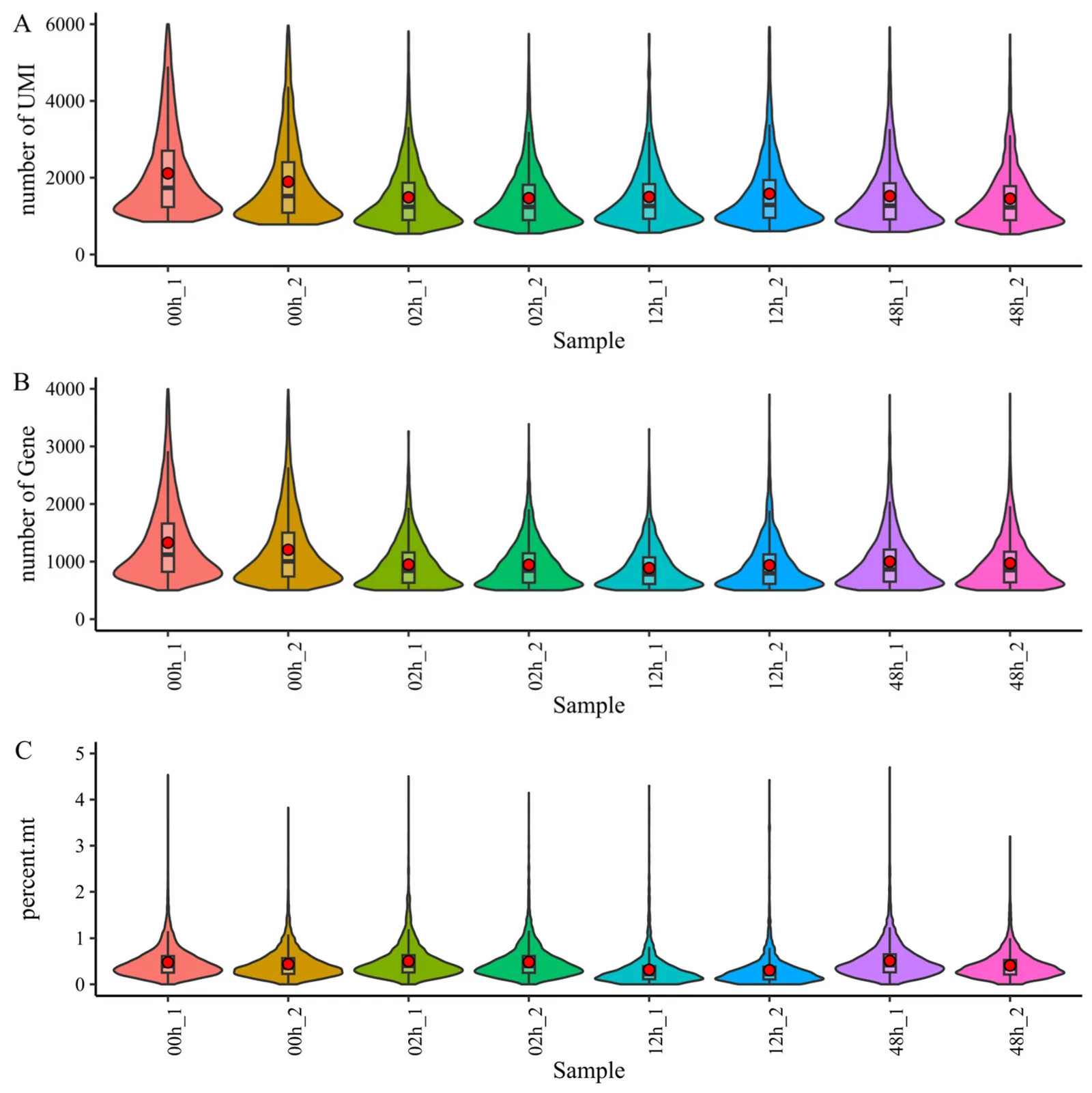
Quality control metrics for single-nucleus RNA sequencing data. (**A**) Violin plots showing the distribution of total UMI (Unique Molecular Identifier) counts per nucleus across samples. (**B**) Violin plots displaying the distribution of total gene counts detected per nucleus across samples. (**C**) Violin plots illustrating the distribution of mitochondrial gene percentage per nucleus across samples.

We initiated our analysis with a comprehensive evaluation of the single-nucleus sequencing data. The assessment revealed that the Q30 base percentages for both barcodes and unique molecular identifiers (UMIs) fell within the range of 92.9% to 94.4%, which is a strong indicator of the sequencing’s exceptional quality. Adhering to the conventional single-cell calling procedure, we meticulously selected high-quality cells for further in-depth analysis. Our selection criteria retained nuclei with 500–40,000 UMI counts and 500–6000 detected genes, while excluding nuclei with a mitochondrial-gene proportion exceeding 10%. This selection criteria helped to exclude the interference from apoptotic or doublet cells, ensuring the dataset’s reliability for subsequent analysis. A total of 32,773 cells were obtained, with distributions as follows: 11,525 cells from the 0 hpi group, 9017 cells from the 2 hpi group, 4121 cells from the 12 hpi group, and 8110 cells from the 48 hpi group (Table 3). The average number of unique reads per cell was between 1460 and 2296 (Table 3 and Figure 4). The median UMI counts per cell were between 1228 and 1785 (Table 3 and Figure 4), while the median gene counts spanned from 780 to 1127 (Table 3 and Figure 4). Notably, the expression patterns of mitochondrial genes indicated that the mitochondrial DNA proportion across all samples was consistently low (<5%) (Figure 4). Collectively, these findings confirm that the single-nucleus RNA sequencing data adheres to the stringent quality control criteria for plant samples, laying a robust groundwork for subsequent analysis.

**Figure 5:**
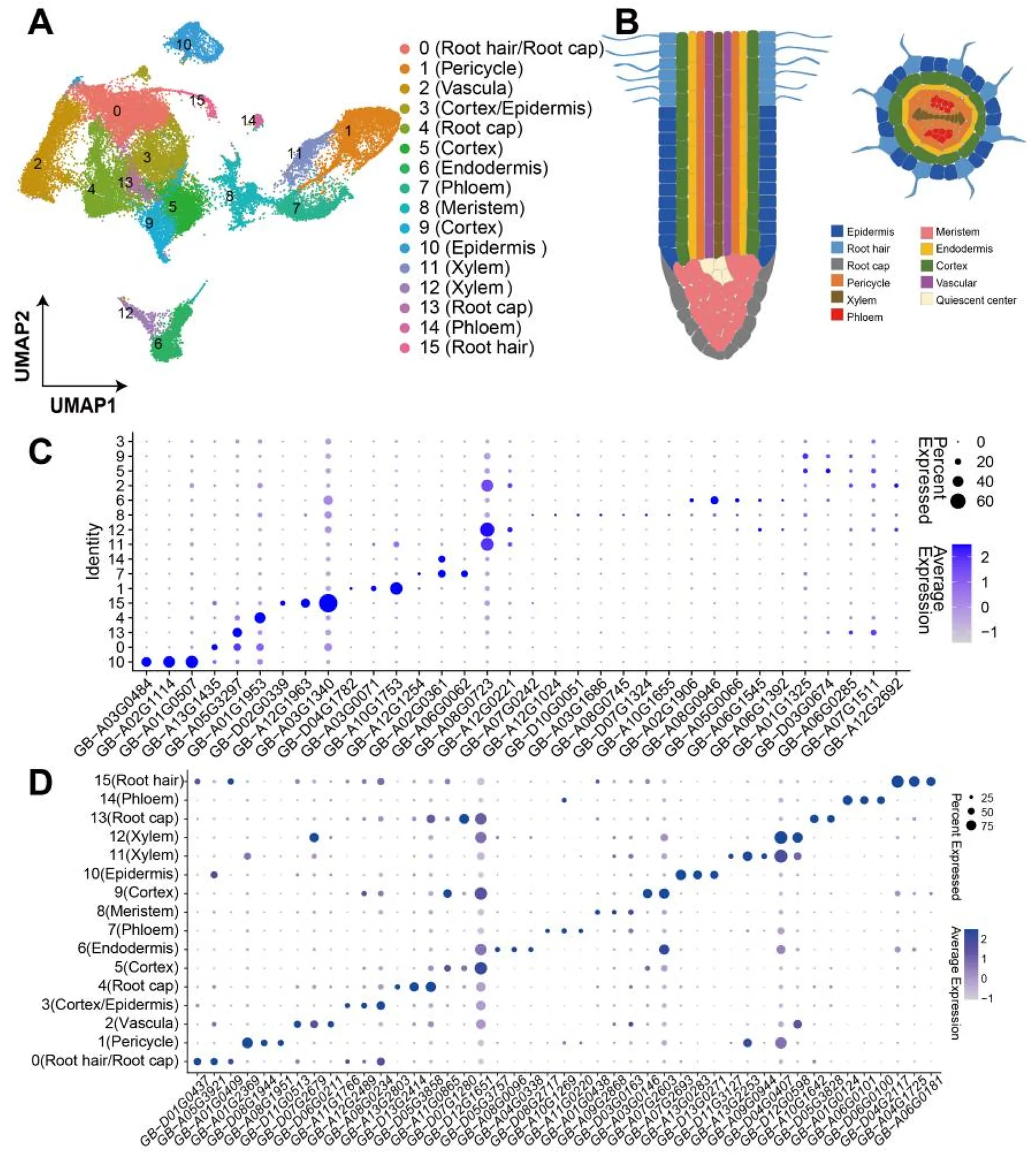
Cell clustering and annotation. (**A**) UMAP dimensional reduction plot showing clustering results and cell type annotations. Each color represents a distinct cell cluster/subpopulation identified through unsupervised clustering analysis. (**B**) A schematic of the cotton root tip structure. (**C**) Representative marker genes used for cell type annotation. (**D**) Dot plot displaying the top 3 differentially expressed marker genes for each cell subpopulation. The size of each dot represents the percentage of cells expressing the gene within each cluster, while the color intensity indicates the average expression level.

Leveraging high-quality single-nucleus data, we constructed the inaugural single-nucleus map of the tetraploid cotton root, resulting in the identification of 16 distinct cell clusters ranging from 310 (cluster 15) to 4378 (cluster 0) (Figure 5A). We utilized marker genes known from diploid cotton and other species in single-cell transcription databases to perform annotation of the tetraploid cotton root cell types; all markers used are listed in Table S1, and representative markers are shown in Figure 5C. Cluster 10 was defined as epidermal cells by markers such as EP1 (GB_A01G0507) [40]; GB_A05G3297, which is homologous to the diploid cotton gene Ga05G3475 [23], was used to define clusters 0 and 13 as the root cap. PER27 (GB_A03G1340) [23,41] was used to define cluster 15 as a root hair type. GB_A12G1963, homologous to diploid cotton’s Ga12G1149 [23], was used to define clusters 0 and 15, which may also be root hair types. argC (GB_A10G1655), UBC19 (GB_D07G1324), and GB_A03G1686 [23,36], which encodes dicer-like protein 2, were used to define cluster 8 as meristematic tissue. IRKI (GB_A08G0946) and MYB36 (GB_A05G0066) [23,42,43] were used to define cluster 6 as the endodermis. ITPK3 (GB_A06G0285) [42] was used to define cluster 2 as vascular tissue. The gene GB_A10G1753, which is homologous to Arabidopsis thaliana’s AT5G43180 [44], was employed to designate cluster 1 as pericycle cells. GB_A02G0361 and GB_A06G0062 were instrumental in identifying clusters 7 and 14 as phloem cells [23,35]. The genes DGAT3 (GB_A11G1099) and MCCA (GB_A08G0723) [45] were utilized as markers to categorize clusters 11 and 12 as xylem tissue types. The gene SIP6 (GB_A01G1325) [41] served as a marker to identify clusters 3, 5, and 9 as cortex tissue types. To further validate the accuracy of our cell type annotations, we applied the Orthologous Marker Gene Groups (OMGs) cross-species annotation method [46], confirming the reliability of key cell-type annotations, including the phloem, meristem, and pericycle (Supplementary Figure S2). To visualize the transcriptional landscape within each cell cluster, we employed a dot plot to illustrate the top three DEGs per cluster (Figure 5D). This representation provides a clear and concise overview of the most dynamically regulated genes, offering insights into the molecular signatures that define each cell type within the root tissue.

### 3.3. Identification of the Key Cell Types Responding to Fov7

**Figure 6:**
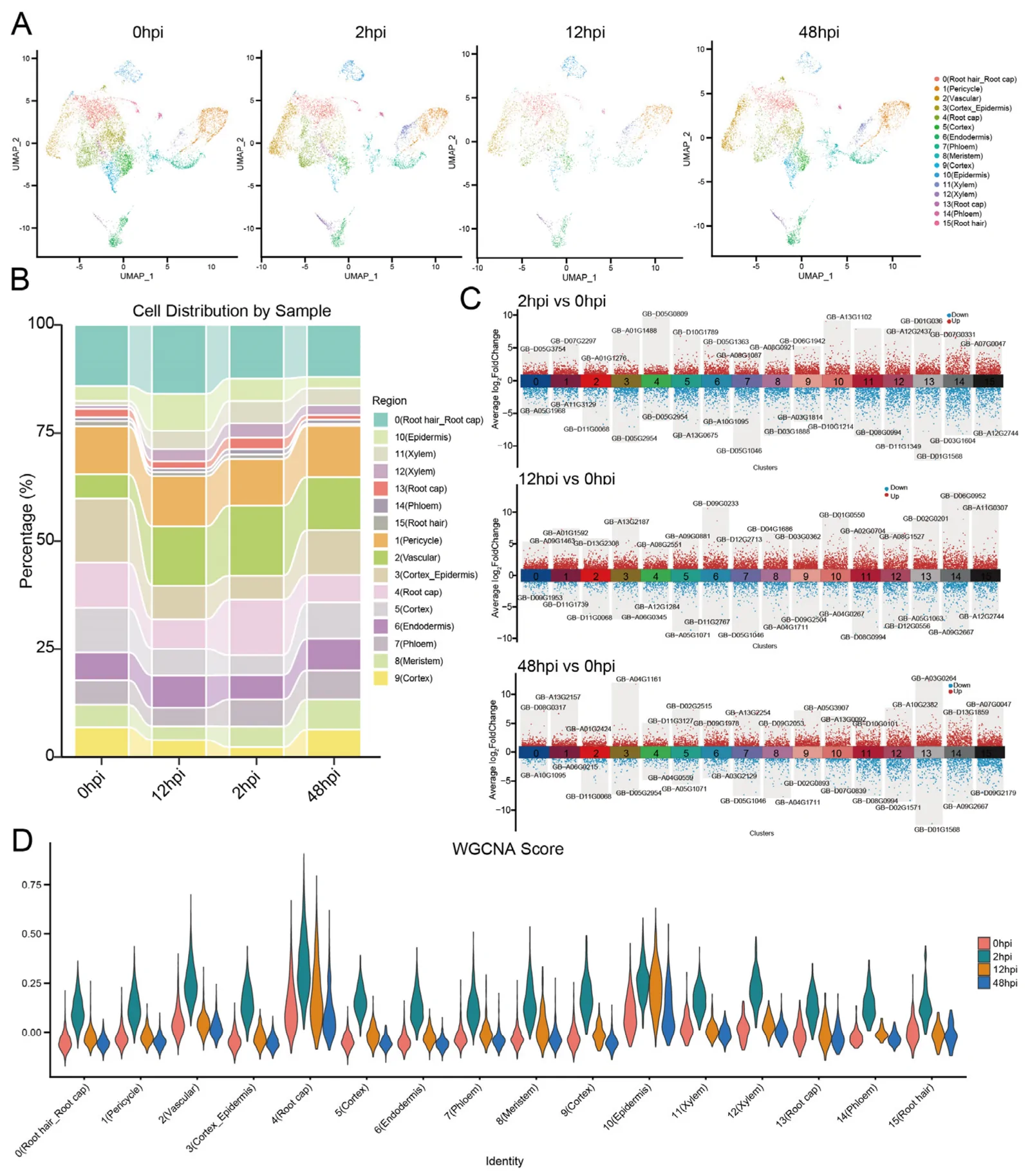
Cell-type-specific responses to Fov7 infection. (**A**) UMAP plot showing the distribution of nuclei from different time points (0, 2, 12, and 48 hpi) after dimensional reduction. (**B**) Stacked bar chart displaying the proportion of nuclei from each time point within different cell types. (**C**) Volcano plot of differential gene expression analysis comparing nuclei from infected time points (2, 12, and 48 hpi) versus uninfected controls (0 hpi) for each cell subpopulation. (**D**) Violin plots showing functional scores based on the 1503 genes identified from WGCNA and K-means intersection analysis.

The UMAP dimensionality reduction analysis revealed consistent cell population distributions across all four time points (0 hpi, 2 hpi, 12 hpi, and 48 hpi) (Figure 6A). The analysis of the percentage composition of various cell types at different treatment time points (0 hpi, 2 hpi, 12 hpi, and 48 hpi) reveals the distribution characteristics of different cell types and their evolutionary trends over time (Figure 6B). For instance, cluster 3 (cortex/epidermis) and cluster 5 (cortex) have higher percentages at the untreated stage (0 hpi), but their proportions gradually decrease as the infection time progresses, whereas cluster 2 (vascular) and cluster 11 (xylem) have the lowest percentages at 0 hpi, but their proportions increase in later treatments. In addition, cluster 4 (Root cap) reaches its peak at 2 hpi, and cluster 10 (epidermis) reaches its peak at 12 hpi (Figure 6B). To reveal the cell-type-specific responses to pathogen infection, we conducted differential gene expression analysis for each cell subpopulation by comparing nuclei from the three infection time points with uninfected controls with the criteria |log_2_FC| > 1 and FDR < 0.05 (Figure 6C). At the 2 h post-inoculation time point, the DEG counts ranged from 515 in cluster 2 (vascular) to 841 in cluster 13 (root cap). At the 12 h post-inoculation time point, the counts were between 412 in cluster 5 (cortex) and 809 in cluster 15 (root hair). By the 48 h post-inoculation time point, the cell counts had shifted to range from 389 in cluster 0 (root hair/root cap) to 925 in cluster 13 (root cap). These variations in cell distribution across different cell types and time points provide valuable insights into the dynamics of cellular response over the course of the experiment. To determine which cell type plays the most critical role in defense responses, we applied a functional scoring approach using the 1503 genes identified from the intersection of K-means cluster C4 and the WGCNA turquoise module (Figure 6D). The analysis showed that cluster 4 (root cap cells) and cluster 10 (epidermis) exhibited the highest functional scores, with peak scores observed at 2 hpi. This finding underscores the biological significance of root cap and epidermis cells in early pathogen recognition and defense initiation, which is consistent with their anatomical position as the first barrier to soil-borne pathogens.

### 3.4. Transcriptional Dynamics of Root Cap and Epidermis Cell Types During Fov7 Infection

Fungal infection is a complex, spatiotemporally dynamic process, with only a subset of plant cells directly interacting with the pathogen [47]. Considering that clusters 4 (root cap) and 10 (epidermis) exhibit high pathogen-associated scores (Figure 6D), we performed trajectory analyses to track their progression through the infection process.

**Figure 7:**
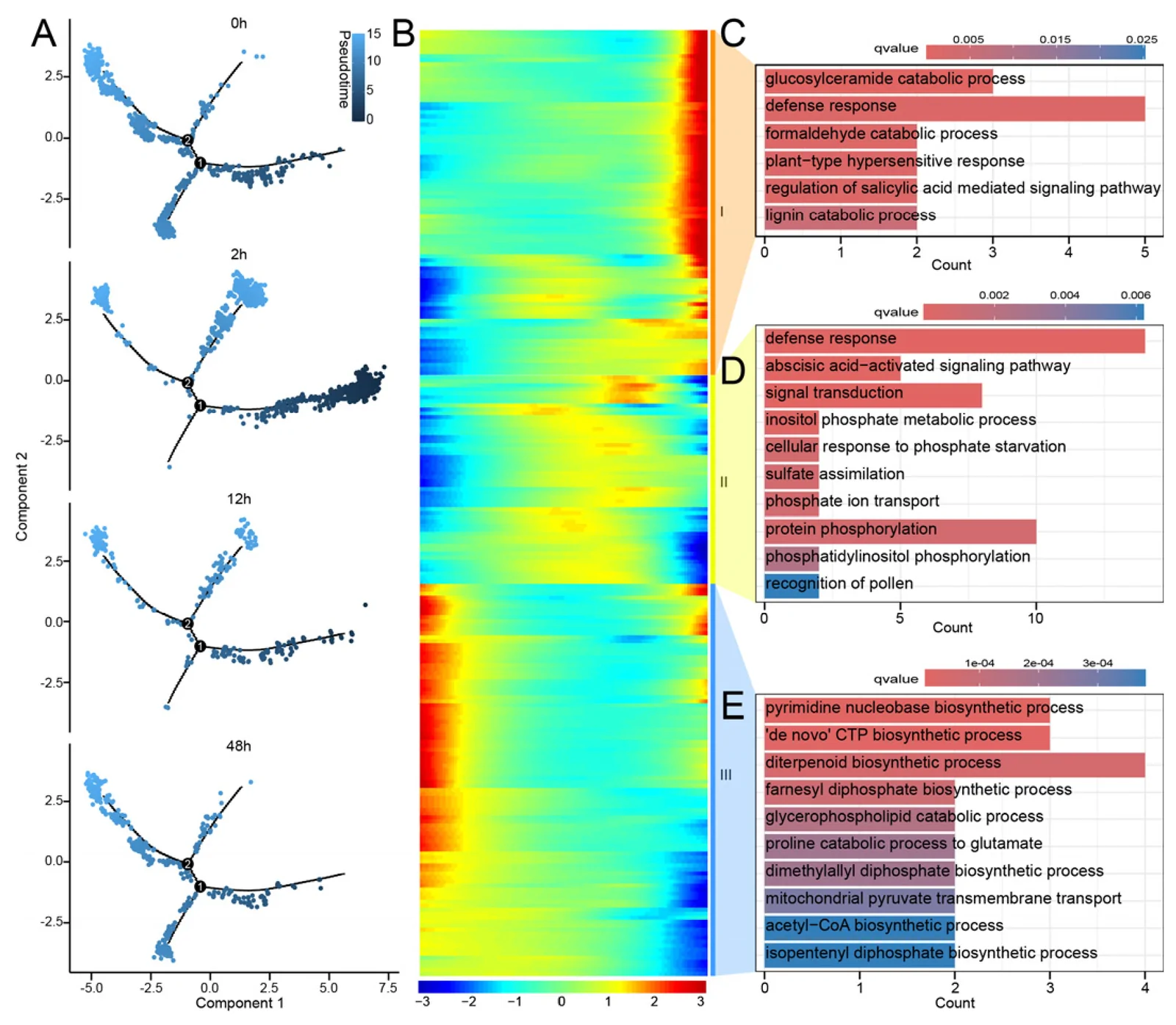
Trajectory analysis of root cap cell type during Fov7 Infection. (**A**) The changes in cell populations in response to Fov7 infection are correlated with the gene expression dynamics induced at infection sites. Trajectory curves of root cap cells from 0 hpi (mock), 2 hpi, 12 hpi, and 48 hpi samples, with each point representing a cell. The color of the points indicates their pseudotime values. (**B**–**E**) Distinct cellular processes are enriched in cell populations with different pseudotime values. The heatmap (**B**) displays the relative expression levels of pseudotime-dependent genes along the root cap cell trajectory, which are divided into three groups based on hierarchical clustering. The most significant Gene Ontology terms enriched in each group (**C**–**E**) are highlighted.

**Figure 8:**
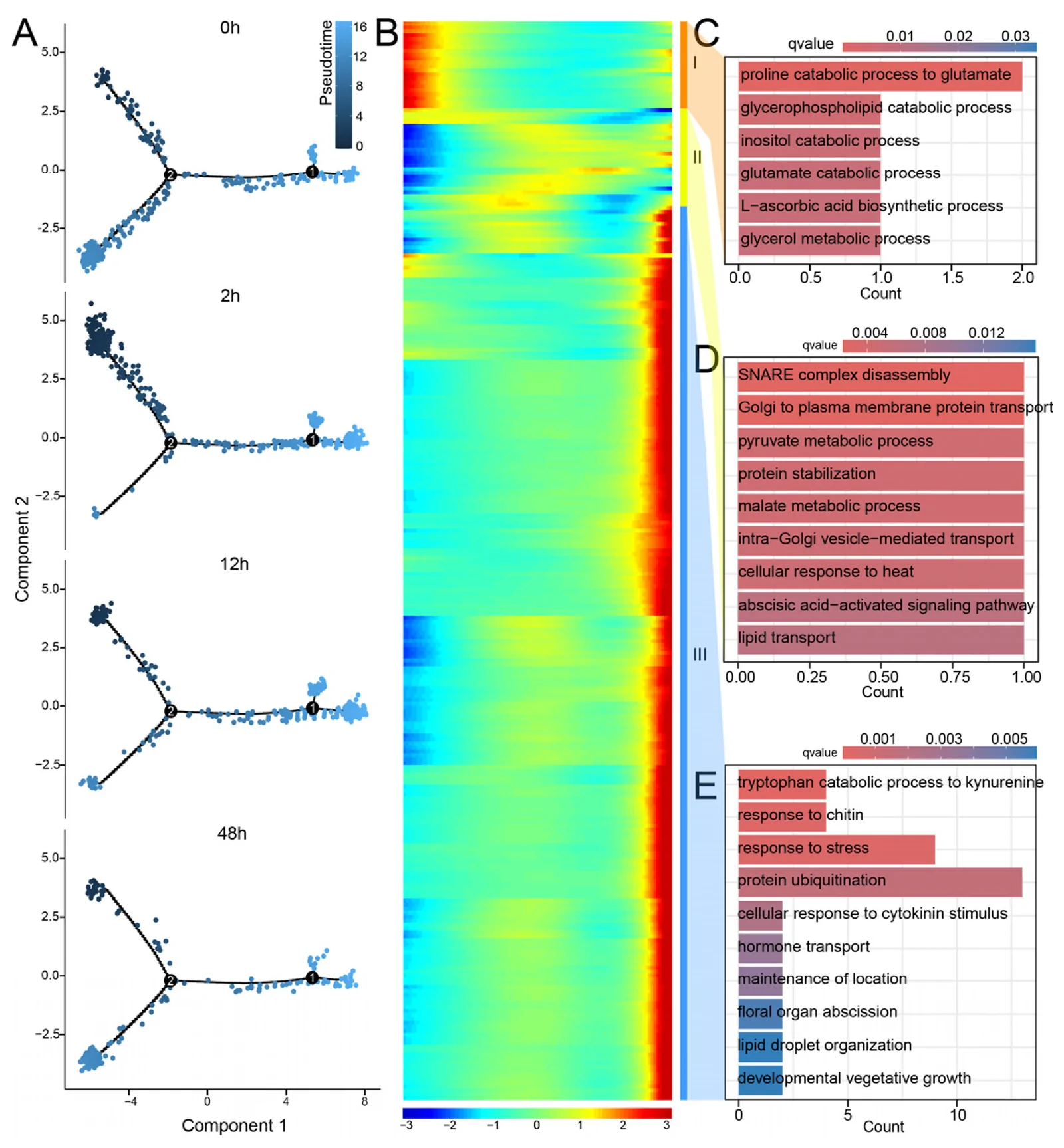
Trajectory analysis of epidermis cell type during Fov7 Infection. (**A**) The changes in cell populations in response to Fov7 infection are correlated with the gene expression dynamics induced at infection sites. Trajectory curves of root cap cells from 0 hpi (mock), 2 hpi, 12 hpi, and 48 hpi samples, with each point representing a cell. The color of the points indicates their pseudotime values. (**B**–**E**) Distinct cellular processes are enriched in cell populations with different pseudotime values. The heatmap (**B**) displays the relative expression levels of pseudotime-dependent genes along the root cap cell trajectory, which are divided into three groups based on hierarchical clustering. The most significant Gene Ontology terms enriched in each group (**C**–**E**) are highlighted.

The first analysis of the root cap (cluster 4) trajectory uncovered 242 genes with pseudotime-dependent expression patterns (Figure 7). Specifically, 56 genes were specifically induced at the end of the trajectory (pseudotime value close to “1”) and GO enrichment analysis revealed an overrepresentation of positive regulators of plant defense at this stage, such as defense response (Figure 7C). This includes four TIR-NBS-LRR genes (GB_A10G2725, GB_A10G2726, GB_D03G1868, and GB_D10G2688) and one PR10 gene (GB_D02G0559), suggesting that immune responses have been activated in these cells. A total of 87 genes were induced in cells midway through the trajectory (Figure 7D), potentially surrounding the infection site without direct contact with fungal hyphae. Another 99 genes were highly expressed at the beginning of the trajectory (pseudotime value close to “0”), with no known functions related to plant defense (Figure 7E). These cells are likely located distal to the fungal infection hyphae (IH) and are minimally affected by the infection, establishing a baseline for our pseudotime analysis. These findings suggest that the transition towards a pseudotime value of “1” implies that the cells have been affected by Fov7 infection and may be in direct contact with IH. Similarly, further examination of the epidermis (cluster 10) trajectory identified 276 genes with pseudotime-dependent changes (Figure 8). At the beginning of the trajectory (pseudotime value close to “0”), 27 genes were highly expressed, again with no known defense-related functions (Figure 8C). These cells are likely distant from the fungal infection hyphae and serve as a reference point for our analysis. An additional 21 genes were induced in cells midway through the trajectory, likely surrounding the infection site, and annotated with one abscisic acid-activated signaling pathway (Figure 8D). As the pseudotime value approaches “1,” signifying the end of the trajectory, 228 genes are specifically activated. GO enrichment analysis uncovered the presence of positive regulators of plant defense responses among these genes, such as those involved in responses to chitin and stress. These include members of the C2H2 genes (GB_A03G0484, GB_A13G0217, GB_A13G2720, GB_D13G2654), ARG2 (GB_A08G0855), DREB2A (GB_A09G1957), SRO3 (GB_D05G2199), SAG21 (GB_D08G0856), and AAA-ATPase (GB_D10G0834) (Figure 8E). The activation of these genes indicates that an immune response has been initiated in these cells. This finding further confirms the association between the shift towards a pseudotime value of “1” and the impact of fungal infection.

### 3.5. RT-qPCR Confirms the Expression Patterns of Key Candidate Genes

**Figure 9:**
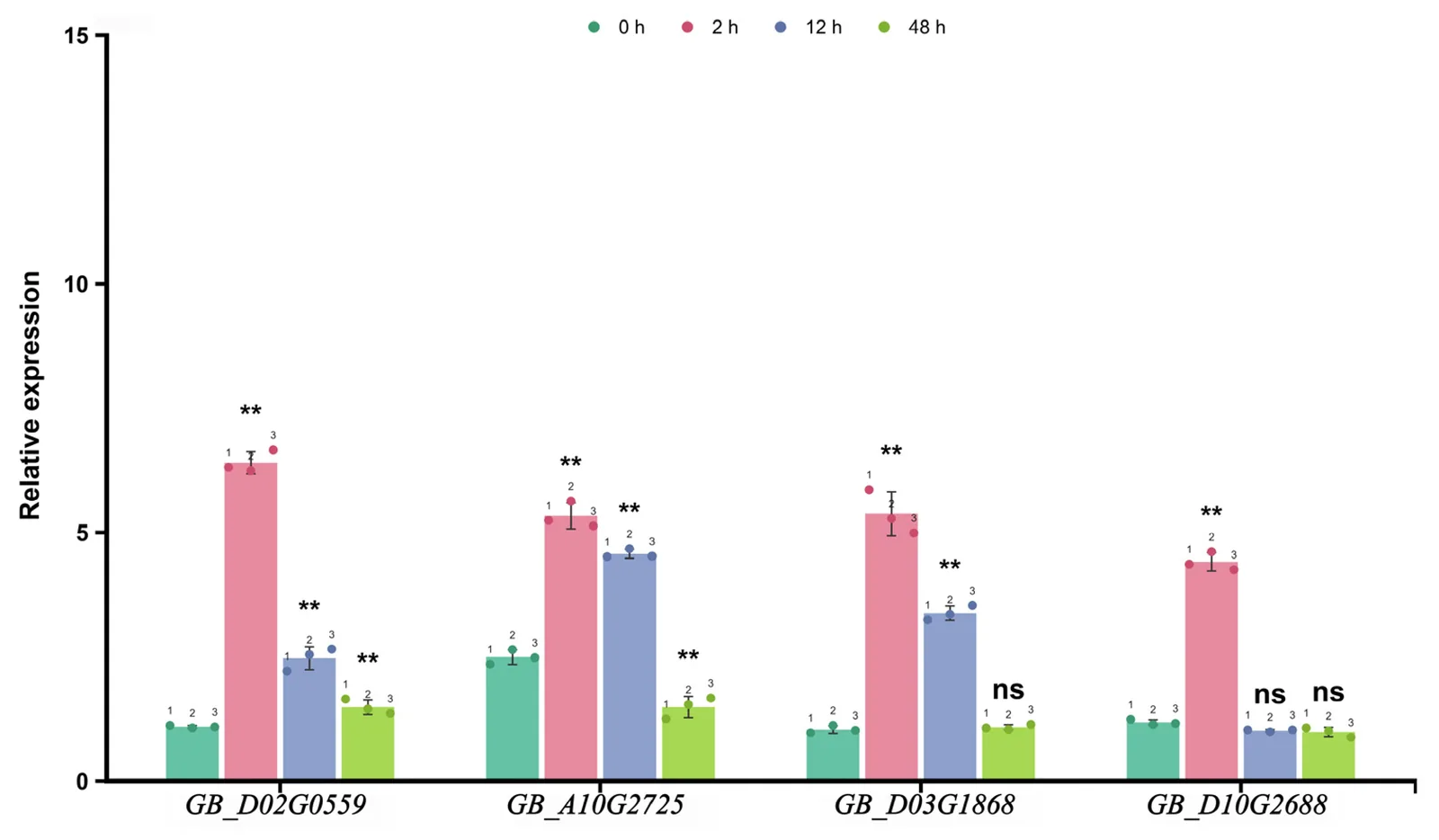
qRT-PCR results of four selected genes under FOV7 infection stress, ** *p* ≤ 0.01.

To corroborate our RNA-seq findings, four candidate genes—one PR10 gene (GB_D02G0559) and three TIR-NBS-LRR genes (GB_A10G2725, GB_D03G1868, GB_D10G2688)—were subjected to RT-qPCR validation. Consistent with our transcriptomic expression profiles, all four genes exhibited significant upregulation at 2 h post-inoculation (hpi) with FOV7, supporting their pivotal roles in the early host defense response against this pathogen (Figure 9).

## 4. Discussion

Time-resolved bulk RNA-seq revealed a pronounced biphasic transcriptional response of *G. barbadense* roots to Fov7. The peak of differential expression at 2 hpi (9230 DEGs) demonstrates that the resistant cultivar undergoes rapid and extensive transcriptomic reprogramming within hours of pathogen perception, aligning with the temporal dynamics of pattern-triggered immunity (PTI) activation in plant–pathogen interactions [48,49]; K-means clustering and weighted gene co-expression network analysis (WGCNA) identified a core set of 1503 genes specifically upregulated at 2 hpi, which exhibited functional enrichment for jasmonic acid (JA)-mediated signaling pathway regulation, wound response, defense response modulation, and abscisic acid (ABA)-activated signaling pathways. The prominence of JA signaling is consistent with the established role of JA as a central defense hormone against necrotrophic and hemibiotrophic pathogens [50,51] and with previous transcriptomic studies in cotton showing JA pathway activation upon Verticillium dahliae and F. oxysporum infection [52,53]. The concurrent enrichment of ABA-activated signaling with JA pathways warrants particular attention; although ABA has traditionally been linked to abiotic stress responses, emerging evidence supports its function as an early defense response modulator in roots [54,55]. The detection of 16 pathogenesis-related protein family member 10 (PR10) genes within the early response cohort holds biological significance, as PR10 proteins are well-characterized pathogen resistance factors possessing documented ribonuclease activity, ligand-binding capacity, and direct antimicrobial properties [42,56]. The subsequent increase in downregulated genes at 12 hpi (7833) likely reflects a metabolic reallocation phase, during which the plant redirects resources from growth toward sustained defense [57]. By 48 hpi, the substantial reduction in DEG number (1653) suggests establishment of a new transcriptional steady state in the resistant genotype, indicative of effective fungal invasion containment rather than the progressive cellular collapse characteristic of compatible interactions.

We present the first single-nucleus transcriptomic atlas of tetraploid cotton (*G. barbadense*) roots during fungal pathogen infection, comprising 32,773 high-quality nuclei resolved into 16 clusters representing ten major cell types. This atlas expands the growing compendium of cotton single-cell studies, which have previously focused on fiber development [16,17,18], anther development [19], terpenoid biosynthesis in glandular trichomes [20], somatic embryogenesis [21,22], and salt stress responses in diploid cotton roots23. Functional scoring using the 1503 core defense genes revealed that root cap (cluster 4) and epidermis (cluster 10) cells exhibited the highest defense scores, peaking at 2 hpi. This finding is anatomically intuitive: root cap and epidermal cells constitute the outermost cell layers that first encounter soil-borne pathogens, positioning them as primary immune sentinels [58,59]. Notably, root cap cells undergo continuous shedding during root growth through soil matrices, with their sloughed border cells secreting mucilage and antimicrobial compounds that function as frontline defense mechanisms [60,61]. The cellular resolution achieved in this study further uncovered temporal dynamics in cell type composition during pathogenesis: cortex cells showed reduced representation while vascular and xylem cells exhibited increased proportions at later infection stages, indicating tissue remodeling processes triggered by *Fusarium oxysporum* f. sp. *vasinfectum* 7 (Fov7). These findings align with emerging evidence that plant roots implement layer-specific defense strategies rather than uniform tissue-wide responses [62,63].

Trajectory inference of root cap (cluster 4) and epidermal (cluster 10) cells revealed ordered, pseudotime-dependent gene expression programs that progress from a basal, non-defensive state to a terminal defense-activated state. In the root cap, 242 pseudotime-dependent genes were partitioned into three groups: 99 early-expressed genes with no established defense functions, 87 intermediately induced genes potentially marking cells proximal to infection sites, and 56 terminally activated genes enriched for defense responses. The terminal defense gene set included four TIR-NBS-LRR (TNL) genes and one PR10 gene. The terminal localization of TNL genes is noteworthy, as TNL-type intracellular immune receptors recognize pathogen effectors delivered into host cells and activate effector-triggered immunity (ETI), a robust defense response often associated with localized cell death that restricts pathogen spread [51,64,65]. In the epidermis, 276 pseudotime-dependent genes followed a similar three-phase pattern: 27 basal genes at initiation, 21 intermediately expressed genes associated with abscisic acid (ABA)-activated signaling pathways, and 228 terminally upregulated genes enriched for chitin responsiveness and stress adaptation. The terminal epidermal gene set included C2H2 zinc-finger transcription factors, ARG2, DREB2A, SRO3, SAG21, and an AAA-ATPase. The enrichment of chitin response genes is particularly notable because chitin is a major fungal cell wall component and a well-characterized microbe-associated molecular pattern (MAMP) recognized by plant LysM-domain receptor complexes to trigger PTI [66,67]. The temporal emergence of ABA-mediated signaling in epidermal cells during the intermediate stage, prior to the complete activation of chitin-responsive defense mechanisms at the terminal phase, indicates that ABA may serve as transitional signal priming cells for subsequent defense activation. This observation aligns with recent single-cell transcriptomic evidence demonstrating ABA’s capacity to non-cell autonomously prime outer root tissues for stress responses [54]. Together, these trajectories provide a cellular-resolution view of the PTI-to-ETI continuum [65], revealing sequential progression of outer root cells from pathogen perception to defense implementation through temporally coordinated molecular programming.

The complementary strengths of bulk RNA-seq and snRNA-seq were systematically exploited in this study: bulk RNA-seq delivered comprehensive transcriptomic profiling with robust temporal resolution across four experimental time points, whereas snRNA-seq enabled deconvolution of cellular heterogeneity and precise localization of defense activation within specific cell types [48,68]. The 1503 core defense genes identified through bulk transcriptomic analysis demonstrated independent validation through their cell-type-specific enrichment patterns as observed in root cap and epidermal tissues using single-cell resolution data, thereby cross-validating the biological relevance of this gene set. This integrative methodology aligns with emerging multiscale approaches in plant pathology that combine bulk and single-cell transcriptomics to elucidate immune responses at different organizational levels [13,47]. For example, single-cell transcriptomics of Arabidopsis leaves infected with Colletotrichum higginsianum revealed cell-type-specific immune responses [13], and single-nucleus transcriptomics of tobacco roots responding to Ralstonia solanacearum uncovered cellular heterogeneity in defense activation [3]. Our study extends this paradigm to cotton, a globally important fiber crop, and to *F. oxysporum* f. sp. *vasinfectum* race 7, the most prevalent and virulent race in China [11]. The generated datasets represent a valuable community resource for future investigations into cotton disease resistance mechanisms, root tissue biology, and immune response evolution of immune responses in polyploid crops.

Several limitations of this study warrant acknowledgment. First, the transcriptomic analyses were performed on a single resistant cultivar; incorporating susceptible genotypes into future single-cell investigations could facilitate direct comparisons of resistance and susceptibility responses at cellular resolution, thereby aiding identification of key determinants differentiating these phenotypes. Second, while our focus centered on Fov7, additional *Fusarium oxysporum* f. sp. *vasinfectum* (Fov) races may induce distinct cellular response patterns, necessitating comparative analyses across multiple races to reveal conserved and race-specific defense mechanisms. Third, while snRNA-seq provides cellular resolution, it lacks spatial context; future spatial transcriptomics approaches, as recently applied in plant roots [54], would help map the physical positions of defense-activated cells relative to fungal hyphae. Fourth, the candidate genes identified here—including TIR-NBS-LRR receptors, C_2_H_2_ zinc-finger transcription factors, and PR10 family members—represent promising candidates for functional validation through reverse genetic approaches such as virus-induced gene silencing or CRISPR-Cas9 editing, which will be necessary to establish causal roles in Fov7 resistance. Despite these constraints, the multi-scale transcriptomic dataset presented here offers a robust framework for elucidating the cellular and molecular mechanisms underlying Fov7 resistance in *Gossypium barbadense*, while providing critical resources to advance marker-assisted breeding of Fusarium wilt-resistant cotton cultivars.

## 5. Conclusions

In this study, we generated an integrated bulk RNA-seq and snRNA-seq resource for Gossypium barbadense roots responding to *Fusarium oxysporum* f. sp. *vasinfectum* race 7 across four time points (0, 2, 12, and 48 hpi). Bulk transcriptomics identified 1503 core early-defense genes that were specifically upregulated at 2 hpi and enriched for jasmonic acid-mediated signaling, wounding response, defense regulation, and abscisic acid-activated signaling, including 16 PR10 family members. Single-nucleus transcriptomics resolved 32,773 nuclei into ten major root cell types and revealed that root cap and epidermis cells function as the primary immune responders during early Fov7 infection. Pseudotime trajectory analysis reconstructed ordered defense activation programs in both the root cap and epidermis, progressing from a basal state through intermediate signaling states to terminal defense execution characterized by TIR-NBS-LRR receptors, chitin-responsive genes, and defense-associated transcription factors. This spatiotemporally resolved molecular atlas provides a high-resolution framework for deciphering cotton-Fusarium interactions at single-cell resolution while establishing a valuable genomic resource for molecular breeding of Fusarium wilt-resistant cotton cultivars.

## Figures and Tables

**Table 1:** Bulk RNA-seq sequencing statistics.

Sample	Read Sum	Base Sum	GC (%)	N (%)	Q20 (%)	Cycle Q20 (%)	Q30 (%)
RC_0h_1	23,317,451	7,041,870,202	44.04	0	99.07	100	97.34
RC_0h_2	22,589,358	6,821,986,116	44.21	0	98.88	100	96.87
RC_0h_3	24,124,008	7,285,450,416	44.47	0	98.93	100	97
RC_2h_1	24,318,746	7,344,261,292	44.84	0.01	98.9	100	96.84
RC_2h_2	22,252,576	6,720,277,952	44.53	0	98.96	100	97.07
RC_2h_3	23,244,127	7,019,726,354	44.68	0	98.95	100	97.04
RC_12h_1	24,770,635	7,480,731,770	44.34	0	98.71	100	96.3
RC_12h_2	24,641,631	7,441,772,562	44.34	0	98.64	100	96.09
RC_12h_3	24,680,622	7,453,547,844	44.33	0	98.72	100	96.32
RC_48h_1	21,184,807	6,397,811,714	44.14	0	98.97	100	97.09
RC_48h_2	21,958,437	6,631,447,974	44.23	0	98.96	100	97.08
RC_48h_3	21,872,583	6,605,520,066	44.15	0	98.94	100	97.03

**Table 2:** Statistics of the snRNA-seq dataset used in this study.

Item	RC_0h_1	RC_0h_2	RC_2h_1	RC_2h_2	RC_12h_1	RC_12h_2	RC_48h_1	RC_48h_2
Sequencing volume (G)	120.0	103.0	100.5	105.1	99.2	98.2	123.9	105.2
Clean reads (M)	400.1	343.4	335.2	350.3	330.7	327.4	413.0	350.7
Valid reads (M)	363.2	305.7	273.8	281.6	229.0	222.0	318.7	263.0
Valid reads (%)	90.8	89.0	81.7	80.4	69.3	67.8	77.2	75.0
Q30 Bases in barcode + UMI (%)	92.9	93.3	93.9	93.6	93.8	94.4	93.8	93.7
Q30 Bases in RNA read (%)	86.0	88.2	86.3	91.0	90.1	88.6	92.4	92.0

**Table 3:** Per-sample quality-control metrics of the snRNA-seq libraries.

Item	RC_0h_1	RC_0h_2	RC_2h_1	RC_2h_2	RC_12h_1	RC_12h_2	RC_48h_1	RC_48h_2
Raw (recovered) nuclei (cell-calling)	6309	6203	6217	6114	5609	5521	5206	5308
Retained nuclei (after QC)	1127	1004	839	841	780	798	864	851
Median genes per nucleus	1354	1210	950	945	888	936	1003	972
Mean genes per nucleus	1785	1542	1242	1239	1262	1298	1271	1228
Median UMI counts per nucleus	2296	1983	1494	1474	1504	1619	1533	1460
Mean UMI counts per nucleus	37,766	36,607	29,812	29,625	22,566	22,989	32,634	31,538
Total genes detected	0	0	0	1	9	6	0	0

## Data Availability

The single-cell RNA sequencing and bulk RNA sequencing raw data utilized in this study can be accessed by the accession numbers CRA025238 and CRA025240, respectively, at National Genomics Data Center, China National Centre for Bioinformation/Beijing Institute of Genomics, Chinese Academy of Science. The RPKM expression matrix data for bulk RNA-seq and RDS (a file format to save Seurat objects) file for single-cell RNA sequencing have been deposited in FigShare (https://doi.org/10.6084/m9.figshare.29467529.v3).

## Supplementary Materials

The following supporting information can be downloaded at: https://www.mdpi.com/article/10.3390/cells15211935/s1.
