# Supplementary material for: Transcriptomic Profiling of Gossypium barbadense Under Fusarium oxysporum f. sp. vasinfectum Race 7 Infection via Bulk RNA-Seq and snRNA-Seq
Source: Cells. 2026 Oct 24;15(21):1935. doi: 10.3390/cells15211935 (PMC13762129; doi:10.3390/cells15211935)
Supplement: Supplementary file 1 [file cells-15-01935-s001.zip › Supplementary Figure S1.pdf]

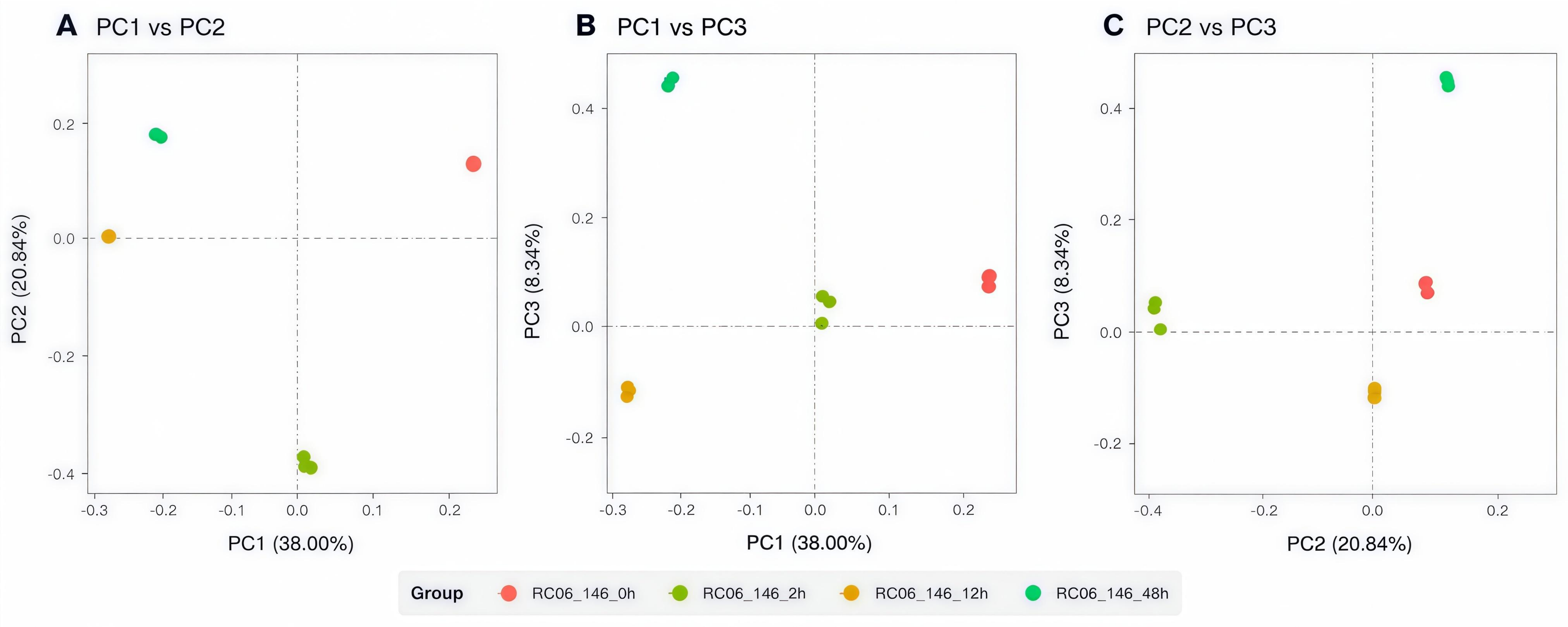

A
PC1 vs PC2
0.2
0.0
-0.2
-0.4
-0.3
-0.2
-0.1
0.0
0.1
0.2
PC1 (38.00%)
PC2 (20.84%)
B
PC1 vs PC3
0.4
0.2
0.0
-0.2
-0.3
-0.2
-0.1
0.0
0.1
0.2
PC1 (38.00%)
PC3 (8.34%)
C
PC2 vs PC3
0.4
0.2
0.0
-0.2
-0.4
-0.2
0.0
0.2
PC2 (20.84%)
PC3 (8.34%)
Group
RC06_146_0h
RC06_146_2h
RC06_146_12h
RC06_146_48h
