# Supplementary material for: Transcriptomic Profiling of Gossypium barbadense Under Fusarium oxysporum f. sp. vasinfectum Race 7 Infection via Bulk RNA-Seq and snRNA-Seq
Source: Cells. 2026 Oct 24;15(21):1935. doi: 10.3390/cells15211935 (PMC13762129; doi:10.3390/cells15211935)

Supplementary Figure S2 Orthologous Marker Gene Groups (OMGs) cross-species annotation.

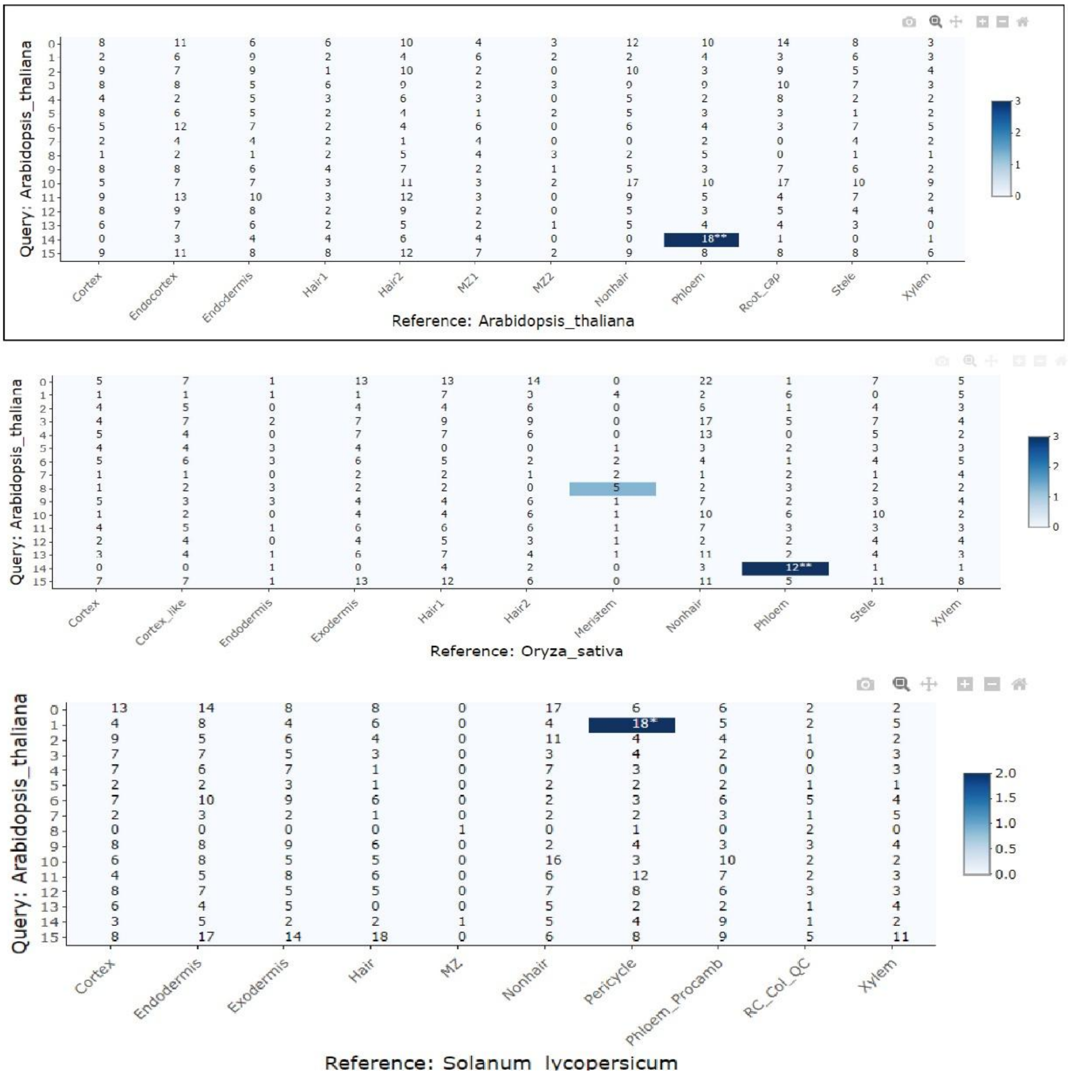

Supplement: Supplementary file 1 [file cells-15-01935-s001.zip › Supplementary Figure S2.pdf]
