# Supplementary material for: Transcriptomic Profiling of Gossypium barbadense Under Fusarium oxysporum f. sp. vasinfectum Race 7 Infection via Bulk RNA-Seq and snRNA-Seq
Source: Cells. 2026 Oct 24;15(21):1935. doi: 10.3390/cells15211935 (PMC13762129; doi:10.3390/cells15211935)
Supplement: Supplementary file 1 [file cells-15-01935-s001.zip › Supplementary Table S1.pdf]

Supplementary Table S1 known marker genes used in this study.

Gene ID	Homologous gene	Homologous species	cell type	source
GB_A01G2359	PVK06_031708	Gossypium arboreum	Epidermis	PlantscRNAdb
GB_A06G0882	PVK06_019618	Gossypium arboreum	Epidermis	PlantscRNAdb
GB_A06G0217	PVK06_018736	Gossypium arboreum	Epidermis	PlantscRNAdb
GB_A05G3463	PVK06_017516	Gossypium arboreum	Epidermis	PlantscRNAdb
GB_A05G3492	PVK06_017464	Gossypium arboreum	Epidermis	PlantscRNAdb
GB_A05G2333	PVK06_015952	Gossypium arboreum	Epidermis	PlantscRNAdb
GB_A05G2284	PVK06_015888	Gossypium arboreum	Epidermis	PlantscRNAdb
GB_A05G1953	PVK06_015518	Gossypium arboreum	Epidermis	PlantscRNAdb
GB_A05G1913	PVK06_015469	Gossypium arboreum	Epidermis	PlantscRNAdb
GB_A05G1433	PVK06_014942	Gossypium arboreum	Epidermis	PlantscRNAdb
GB_A01G1011	PVK06_014685	Gossypium arboreum	Epidermis	PlantscRNAdb
GB_A05G0627	PVK06_014073	Gossypium arboreum	Epidermis	PlantscRNAdb
GB_A05G0510	PVK06_013953	Gossypium arboreum	Epidermis	PlantscRNAdb
GB_A05G0174	PVK06_013599	Gossypium arboreum	Epidermis	PlantscRNAdb
GB_A05G3854	PVK06_011064	Gossypium arboreum	Epidermis	PlantscRNAdb
GB_A03G1166	PVK06_008274	Gossypium arboreum	Epidermis	PlantscRNAdb
GB_A03G2129	PVK06_006768	Gossypium arboreum	Epidermis	PlantscRNAdb
GB_A02G1791	PVK06_005984	Gossypium arboreum	Epidermis	PlantscRNAdb
GB_A02G1212	PVK06_004709	Gossypium arboreum	Epidermis	PlantscRNAdb
GB_A01G1917	PVK06_004523	Gossypium arboreum	Epidermis	PlantscRNAdb
GB_A03G0484	PVK06_003104	Gossypium arboreum	Epidermis	PlantscRNAdb
GB_A02G1114	PVK06_002369	Gossypium arboreum	Epidermis	PlantscRNAdb
GB_A01G0792	PVK06_000920	Gossypium arboreum	Epidermis	PlantscRNAdb
GB_A01G0725	PVK06_000847	Gossypium arboreum	Epidermis	PlantscRNAdb
GB_A01G0507	PVK06_000576	Gossypium arboreum	Epidermis	PlantscRNAdb
GB_A01G0507	Ga01G0569	Gossypium arboreum	Epidermis	Li et al., 2023
GB_A06G2363	Ga06G2370	Gossypium arboreum	Root cap	Li et al., 2023
GB_A05G3297	Ga05G3475	Gossypium arboreum	Root cap	Li et al., 2023
GB_A08G1947	Ga08G1867	Gossypium arboreum	Root cap	Li et al., 2023
GB_A13G2470	Ga14G0307	Gossypium arboreum	Root cap	Li et al., 2023
GB_A01G1953	PVK06_004473	Gossypium arboreum	Root cap	PlantscRNAdb
GB_D06G0980	Ga06G0968	Gossypium arboreum	Root hair	Li et al., 2023
GB_A10G0547	Ga10G2506	Gossypium arboreum	Root hair	Li et al., 2023
GB_D02G0339	Ga14G2589	Gossypium arboreum	Root hair	Li et al., 2023
GB_A12G1963	Ga12G1149	Gossypium arboreum	Root hair	Li et al., 2023
GB_A03G1340	Ga03G1501	Gossypium arboreum	Root hair	Li et al., 2023
GB_D04G1782	AT3G25190	Arabidopsis thaliana	Pericycle	PlantscRNAdb
GB_A03G0071	AT1G32450	Arabidopsis thaliana	Pericycle	PlantscRNAdb
GB_A10G1753	AT5G43180	Arabidopsis thaliana	Pericycle	PCMDB
GB_A03G0071	AT3G25190	Arabidopsis thaliana	Pericycle	PCMDB
GB_A03G2293	Ga03G2545	Gossypium arboreum	Phloem	Li et al., 2023
GB_D13G1158	Ga13G1135	Gossypium arboreum	Phloem	Li et al., 2023
GB_A08G0234	AT2G18960	Arabidopsis thaliana	Phloem	PlantscRNAdb
GB_A05G2783	PVK06_016484	Gossypium arboreum	Phloem	PlantscRNAdb
GB_A02G0361	PVK06_009828	Gossypium arboreum	Phloem	PlantscRNAdb
GB-A06G0062	Ga06G0071	Gossypium arboreum	Phloem	Li et al., 2023
GB_A03G1937	PVK06_007004	Gossypium arboreum	Phloem	PlantscRNAdb
GB_A11G1099	AT1G48300	Arabidopsis thaliana	Xylem	PlantscRNAdb
GB_A08G0723	AT1G03090	Arabidopsis thaliana	Xylem	PlantscRNAdb
GB_A07G0242	Ga07G0262	Gossypium arboreum	Meristem	Li et al., 2023
GB_A12G1024	Ga12G2102	Gossypium arboreum	Meristem	Li et al., 2023
GB_D10G0051	Ga10G3048	Gossypium arboreum	Meristem	Li et al., 2023
GB_A03G1686	Ga03G1871	Gossypium arboreum	Meristem	Li et al., 2023
GB_A08G0745	Ga08G0783	Gossypium arboreum	Meristem	Li et al., 2023
GB_D07G1324	Ga07G1357	Gossypium arboreum	Meristem	Li et al., 2023
GB_A10G1655	Ga10G1739	Gossypium arboreum	Meristem	Li et al., 2023

GB_A05G0190	PVK06_013617	Gossypium arboreum	Meristem	PlantscRNAdb
GB_A04G1586	PVK06_013128	Gossypium arboreum	Meristem	PlantscRNAdb
GB_A01G2007	PVK06_004408	Gossypium arboreum	Meristem	PlantscRNAdb
GB_A06G1301	PVK06_020280	Gossypium arboreum	Meristem	PlantscRNAdb
GB_A02G0431	PVK06_009734	Gossypium arboreum	Meristem	PlantscRNAdb
GB_A04G1613	PVK06_013163	Gossypium arboreum	Meristem	PlantscRNAdb
GB_A03G2201	PVK06_006690	Gossypium arboreum	Meristem	PlantscRNAdb
GB_A05G4306	PVK06_010468	Gossypium arboreum	Meristem	PlantscRNAdb
GB_A01G0915	PVK06_001071	Gossypium arboreum	Meristem	PlantscRNAdb
GB_A01G0079	PVK06_000080	Gossypium arboreum	Meristem	PlantscRNAdb
GB_A03G2175	PVK06_006719	Gossypium arboreum	Meristem	PlantscRNAdb
GB_A04G0408	PVK06_000252	Gossypium arboreum	Meristem	PlantscRNAdb
GB_A05G2833	PVK06_035922	Gossypium arboreum	Meristem	PlantscRNAdb
GB_A06G0374	PVK06_018921	Gossypium arboreum	Meristem	PlantscRNAdb
GB_A04G0032	PVK06_048609	Gossypium arboreum	Meristem	PlantscRNAdb
GB_A06G2222	PVK06_021795	Gossypium arboreum	Meristem	PlantscRNAdb
GB_A02G0526	PVK06_009602	Gossypium arboreum	Meristem	PlantscRNAdb
GB_A02G0620	PVK06_009480	Gossypium arboreum	Meristem	PlantscRNAdb
GB_A01G0152	PVK06_000149	Gossypium arboreum	Meristem	PlantscRNAdb
GB_A01G2369	PVK06_003946	Gossypium arboreum	Meristem	PlantscRNAdb
GB_A02G0266	PVK06_009940	Gossypium arboreum	Meristem	PlantscRNAdb
GB_A01G2356	PVK06_003964	Gossypium arboreum	Meristem	PlantscRNAdb
GB_A04G0296	PVK06_018100	Gossypium arboreum	Meristem	PlantscRNAdb
GB_A05G3970	PVK06_010909	Gossypium arboreum	Meristem	PlantscRNAdb
GB_A02G0218	PVK06_010019	Gossypium arboreum	Meristem	PlantscRNAdb
GB_A01G0271	PVK06_000273	Gossypium arboreum	Meristem	PlantscRNAdb
GB_A05G0055	PVK06_013465	Gossypium arboreum	Meristem	PlantscRNAdb
GB_A05G1644	PVK06_015186	Gossypium arboreum	Meristem	PlantscRNAdb
GB_A05G0500	PVK06_013940	Gossypium arboreum	Meristem	PlantscRNAdb
GB_A03G2273	PVK06_006612	Gossypium arboreum	Meristem	PlantscRNAdb
GB_A03G0868	PVK06_002536	Gossypium arboreum	Meristem	PlantscRNAdb
GB_A02G0278	PVK06_009929	Gossypium arboreum	Meristem	PlantscRNAdb
GB_A05G4381	PVK06_010377	Gossypium arboreum	Meristem	PlantscRNAdb
GB_A05G1052	PVK06_014517	Gossypium arboreum	Meristem	PlantscRNAdb
GB_A03G0798	PVK06_002639	Gossypium arboreum	Meristem	PlantscRNAdb
GB_A12G1455	Ga12G1686	Gossypium arboreum	Endodermis	Li et al., 2023
GB_A02G1829	Ga02G0251	Gossypium arboreum	Endodermis	Li et al., 2023
GB_A11G2291	Ga11G1771	Gossypium arboreum	Endodermis	Li et al., 2023
GB_A02G1906	Ga02G0198	Gossypium arboreum	Endodermis	Li et al., 2023
GB_A08G0946	Ga08G0971	Gossypium arboreum	Endodermis	Li et al., 2023
GB_A05G0066	Ga05G0063	Gossypium arboreum	Endodermis	Li et al., 2023
GB_A07G1309	Ga07G1336	Gossypium arboreum	Endodermis	Li et al., 2023
GB_A05G0995	PVK06_014462	Gossypium arboreum	Endodermis	PlantscRNAdb
GB_A05G0066	PVK06_013477	Gossypium arboreum	Endodermis	PlantscRNAdb
GB_A04G1406	PVK06_012908	Gossypium arboreum	Endodermis	PlantscRNAdb
GB_A04G1218	PVK06_012656	Gossypium arboreum	Endodermis	PlantscRNAdb
GB_A05G4039	PVK06_010825	Gossypium arboreum	Endodermis	PlantscRNAdb
GB_A05G4332	PVK06_010436	Gossypium arboreum	Endodermis	PlantscRNAdb
GB_A02G0406	PVK06_009767	Gossypium arboreum	Endodermis	PlantscRNAdb
GB_A03G1269	PVK06_008138	Gossypium arboreum	Endodermis	PlantscRNAdb
GB_A02G1883	PVK06_006108	Gossypium arboreum	Endodermis	PlantscRNAdb
GB_A02G1680	PVK06_005683	Gossypium arboreum	Endodermis	PlantscRNAdb
GB_A01G2128	PVK06_004244	Gossypium arboreum	Endodermis	PlantscRNAdb
GB_A01G2248	PVK06_004095	Gossypium arboreum	Endodermis	PlantscRNAdb
GB_A01G1247	PVK06_001647	Gossypium arboreum	Endodermis	PlantscRNAdb
GB_A01G1923	PVK06_004516	Gossypium arboreum	Cortex	PlantscRNAdb
GB_A01G1056	PVK06_001290	Gossypium arboreum	Cortex	PlantscRNAdb
GB_A13G0219	AT3G55120	Arabidopsis thaliana	Cortex	PlantscRNAdb

GB_A05G3855	PVK06_011062	Gossypium arboreum	Cortex	PlantscRNAdb
GB_D11G0250	AT4G12510	Arabidopsis thaliana	Cortex	PlantscRNAdb
GB_A03G0131	PVK06_003534	Gossypium arboreum	Cortex	PlantscRNAdb
GB_A09G2667	AT5G49360	Arabidopsis thaliana	Cortex	PlantscRNAdb
GB_D03G0674	Ga02G0624	Gossypium arboreum	Cortex	*Li et al., 2023*
GB_A05G3776	Ga04G1402	Gossypium arboreum	Cortex	*Li et al., 2023*
GB_A06G0285	Ga14G1479	Gossypium arboreum	Vascular	*Li et al., 2023*
GB_A07G1511	Ga07G1530	Gossypium arboreum	Vascular	*Li et al., 2023*
GB_A12G2692	Ga12G0396	Gossypium arboreum	Vascular	*Li et al., 2023*
GB_A01G1325	AT3G57520	Arabidopsis thaliana	Cortex	PlantscRNAdb
